# Supplementary material for: Complex‐centric proteome profiling by SEC‐SWATH‐MS
Source: Mol Syst Biol. 2019 Jan 14;15(1):e8438. doi: 10.15252/msb.20188438 (PMC6346213; doi:10.15252/msb.20188438)
Supplement: Supplementary file 8 — Dataset EV7 [file MSB-15-e8438-s008.zip › feature_plots_string/Q7L3B6.pdf]

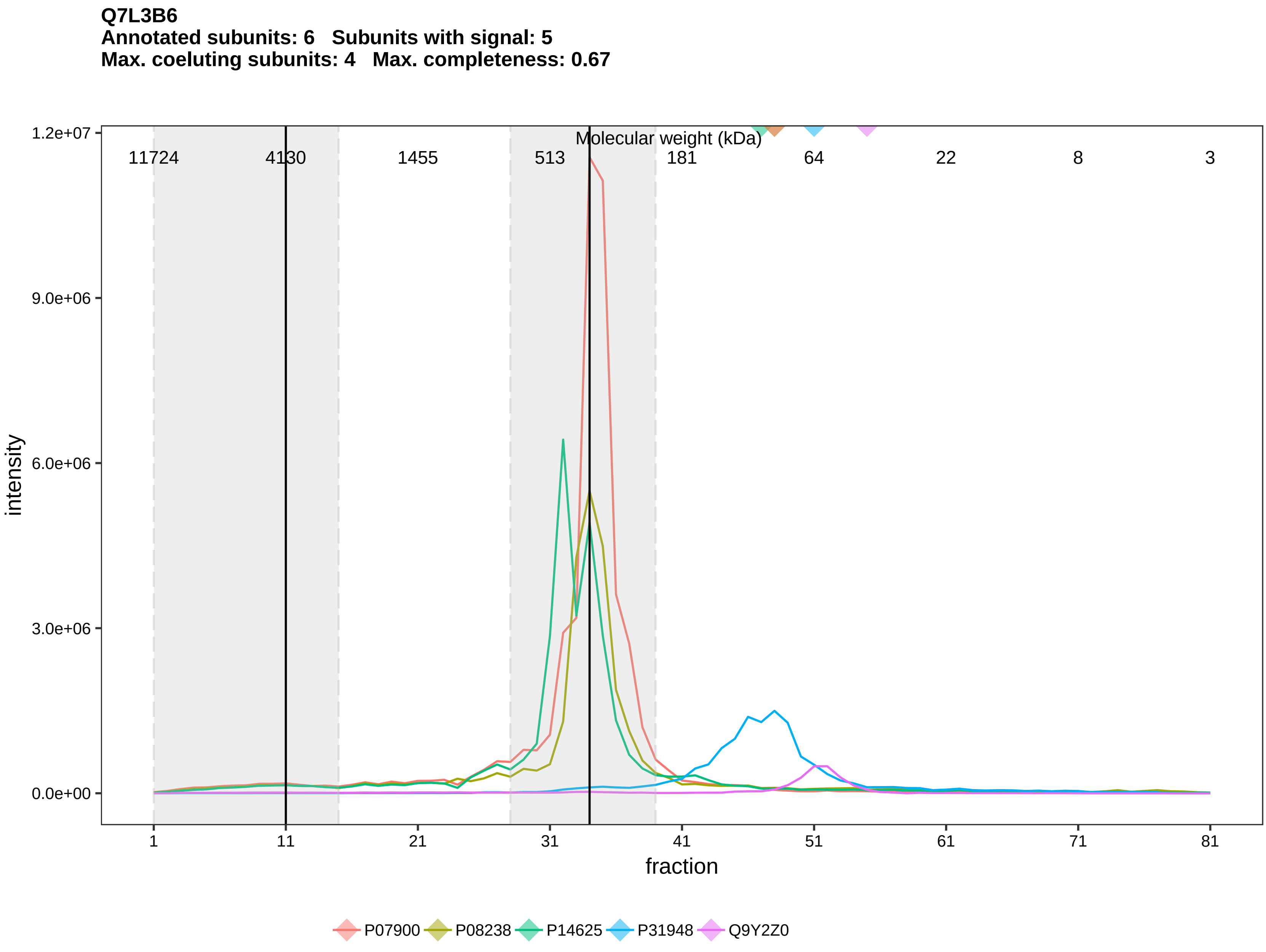
Q7L3B6
Annotated subunits: 6   Subunits with signal: 5
Max. coeluting subunits: 4   Max. completeness: 0.67
Molecular weight (kDa)
11724
4130
1455
513
181
64
22
8
3
1.2e+07
9.0e+06
6.0e+06
3.0e+06
0.0e+00
intensity
1
11
21
31
41
51
61
71
81
fraction
P07900
P08238
P14625
P31948
Q9Y2Z0
